# Do children’s previous physical activity habits influence their behaviors during the Covid-19 social distancing period?

**DOI:** 10.1590/1984-0462/2022/40/2021010

**Published:** 2022-01-05

**Authors:** Cristhina Bonilha Huster Siegle, André Pombo, Carlos Luz, Luis Paulo Rodrigues, Rita Cordovil, Cristina dos Santos Cardoso de Sá

**Affiliations:** a Universidade Federal de São Paulo, Santos, SP, Brazil.; b Universidade de Lisboa, Cruz-Quebrada, Portugal.; c Instituto Politécnico de Lisboa, Lisboa, Portugal.; d Instituto Politécnico de Viana do Castelo, Viana do Castelo, Portugal.

**Keywords:** Coronavirus infection, Motor activity, Child development, Pandemic, Social isolation, Infecções por coronavírus, Atividade motora, Desenvolvimento infantil, Pandemia, Isolamento social

## Abstract

**Objective:**

Verify whether the practice of physical activity, before the social distancing imposed by COVID-19, influences children’s routines during this period, in children of different ages.

**Methods:**

Descriptive cross-sectional study carried out with an online questionnaire from LimeSurvey and disseminated for four months during social distancing. The questionnaire contained questions about family composition, household characteristics, household and children’s routines, including habits such as sleeping, physical activity, intellectual activity, playing with and without physical activity, and screen time. The final sample consisted of 916 participating families that answered about the physical activity habits of their respective children before the pandemic. Children were divided into three age groups (three to five years, six to nine years, and ten to twelve years). Independent Student’s *t*-tests were performed to investigate whether the previous practice of scheduled physical activity group and the no physical activity group differed as to the time dedicated to children’s activities and routines (intellectual activity, sleeping, screen time, playing with and without physical activity), by age groups, during social distancing.

**Results:**

There was a decrease in the levels of physical activity undertaken by Brazilian children during social distancing. There was no difference when both groups. The children with previous practice of scheduled physical activity did not display different habits from the children who did not adopt this practice.

**Conclusions:**

The practice of physical activity before social distancing did not influence the level of physical activity and other habits during social distancing. Healthy habits should be encouraged and targeted for all children during the pandemic. These findings can contribute to the formulation of public policies for children during pandemic times.

## Resumo

**Objetivo:**

Verificar se a prática de atividade física antes do distanciamento social imposto pela COVID-19 influencia a rotina de crianças de diferentes faixas etárias durante esse período.

**Métodos:**

Estudo transversal descritivo realizado com base em um questionário online do LimeSurvey e divulgado por quatro meses durante o distanciamento social. O questionário continha perguntas sobre a composição familiar, características domiciliares, rotinas domésticas e infantis, incluindo hábitos como sono, atividade física, atividade intelectual, lazer com e sem atividade física e tempo de tela. Participaram da amostra final 916 famílias, as quais responderam sobre os hábitos de atividade física de seus filhos antes da pandemia. As crianças foram divididas em 3 faixas etárias (3–5 anos, 6–9 anos e 10–12 anos). Testes *t* de Student independentes foram realizados para investigar se os grupos que praticavam e não praticavam atividade física programada anteriormente diferiam quanto ao tempo dedicado às atividades e rotinas infantis (atividade intelectual, sono, tempo de tela, lazer com e sem atividade física), por faixa etária, durante o distanciamento social.

**Resultados:**

Houve diminuição da atividade física de crianças brasileiras durante o distanciamento social. Não houve diferença entre os grupos. Crianças que praticavam atividade física programada anteriormente não apresentaram hábitos diferentes daquelas que não adotavam essa prática.

**Conclusões:**

A prática de atividade física antes do distanciamento social não influenciou o nível de atividade física e outros hábitos das crianças durante esse período. Hábitos saudáveis devem ser estimulados e direcionados a todas as crianças ao longo da pandemia. Esses achados podem contribuir para a elaboração de políticas públicas para crianças em períodos de pandemia.

## INTRODUCTION

The coronavirus pandemic (COVID-19) was declared on March 11, 2020, with social distancing measures being taken worldwide to slow virus spread.^1–4^ Most of the people infected develop a mild or moderate respiratory infection, recovering without the need for special treatment. However, some people develop severe acute respiratory syndrome.^5^ To control the disease, populations in different countries were instructed to avoid attending public places. Schools, daycare centers, clubs, universities, parks, and non-essential services were closed.^1,2,5^ This kind of movement restriction can impact the maintenance of healthy habits, in addition to generating environmental and social changes.^1,2^ It is the opposite of what happens when children attend school, when they have a more structured routine, leading to healthier behaviors concerning the practice of physical activity (PA), amount of sleep, and healthy food habits. The lack of a structured routine, which impairs the practice of regular physical activity, influences other habits, such as the amount of sleep and screen time, for example^6^. Studies have shown that soon after social distancing measures had been taken in different countries, there was a decrease in PA and an increase in sedentarism.^7–9^

Maintaining an adequate level of PA is important to improve physiological functions and maintain quality of life, as well as to reduce the incidence of viral infections, mental illnesses, and chronic diseases, such as obesity and diabetes.^1,3–5,10^ PA habits are crucial for the development and growth of children.^11,12^ PA improves children’s motor skills, muscular strength, flexibility, and coordination, in addition to being an important resource to increase children’s resilience, problem-solving capacity, emotional well-being, and social interaction.^12,13,14^ Also, lifelong healthy behaviors are formed and consolidated during childhood, with a tendency to maintain, in adulthood, PA levels existing during childhood.^7,12^

Studies show that worldwide, the level of daily PA recommendation is no longer reached by the majority of the population, whether adults or children.^2–4^ A study with 356 Brazilian children, from 5 to 9 years old, classified 38.2% of them as inactive, performing less than 60 minutes of PA per day.^15^ The COVID-19 pandemic and social distancing further influenced healthy habits maintenance,^2–5^ such as the PA undertaken by children and adolescents,^1,5,11^ possibly due to the widespread closure of PA and leisure venues,^2^ such as playgrounds, schools, and other recreational centers,^4^ as well as the suspension of sports training.^3^ However, little is known about the factors that can influence the maintenance of this habit during social isolation and whether the existence of healthy childhood habits before social distancing can help to maintain a higher level of PA during this period.

Thus, the objective of this study was to verify whether the habit of physical activity before the moment of social distancing imposed by COVID-19 influences children’s routines during this period, in children of different age groups.

## METHOD

A descriptive cross-sectional study design using an anonymous online survey was launched to assess how Brazilian families with children under 13 years of age adjusted their daily routines to social distancing. The sample consisted of Brazilian children aged 3 to 12 years and their families, who were in a situation of social distancing due to the COVID-19 pandemic.

This research study is part of an international research study from Universidade de Lisboa (UL). To assess how families with children are dealing with the lockdowns imposed by COVID-19, a questionnaire was created on LimeSurvey online software hosted at Faculdade de Motricidade Humana – UL. The questionnaire was drafted by a committee of experts in the field, and tested in 15 families (pre-test). It was published after some adjustments to the layout of responses regarding the number of hours of activities performed by children. The research was approved by the Research Ethics Committee of Universidade Federal de São Paulo (Certificate of Submission for Ethical Consideration [CAAE] 30930120.2.000.5505, No. 0413/2020).

In Brazil, the questionnaire was launched online on March 24, 2020, and disseminated on social media (Facebook, Instagram, WhatsApp) and via email, following the snowball sampling technique. The questionnaire is anonymous, takes five minutes to complete, and has four sections:

- Family: family composition, number of children and adults in the household, and how many of them are doing their professional activity or working from home.
- Household characteristics: type and characteristics of the household, whether or not there is indoor and outdoor space for PA.
- Household routines: level of concern regarding COVID-19 and how family routines are being adjusted (PA time, screen time, sleep, family activities).
- Children’s routines: characterization of each child (age, sex, and health status), number of hours spent doing different activities in the previous day, and a question about performing physical activity at least twice a week before the period of social distancing.

The questionnaires completed by parents/guardians of all children under 13 years of age living in the same residence, from March 25 to July 24, 2020, during social distancing, were included in this study, totalizing 1,677 responses. All participants read information about the study and agreed to the conditions, clicking to proceed on the first page of the survey. They were allowed to withdraw at any time, by not continuing to fill in the survey or not sending information. After cleaning the database, responses related to 916 children aged 3 to 12 years (474 boys and 442 girls) were included in this study. Responses from 761 children (45.4% of the 1,677 children initially reported) were excluded due to missing or obviously wrong information (for example, a respondent reported more than 24 hours in a day or no sleep time reported for children). Children were divided into three age groups (G1=3–5 years, n=324; G2=6–9 years, n=402, and G3=10–12 years, n=190), and in two groups regarding their previous practice of scheduled physical activity (PPSPA). Children with previous practice (i.e, those who performed PPSPA at least twice a week, before the social distancing period), or without previous practice (i.e., those who performed PPSPA less than twice a week, before the social distancing period).

Descriptive statistics and frequency analysis were used to describe the environments and routines of the family and children during this period. Six categories of activities were analyzed: Intellectual activity (school assignments and online classes), playful screen time (games, movies, social media, Internet, videos, and voice calls), playtime without PA (reading, drawing, painting, board games, etc.), playtime with PA (running, jumping, playing hide-and-seek, etc.), PA (planned, inside and outside the house, walking a dog), and sleep time. The categories intellectual activity, playful screen time, and playtime without PA were grouped to calculate the overall sedentary time, and the categories playtime with PA and PA were grouped to calculate the overall PA time.

Independent Student’s *t*-tests were performed to investigate if the PPSPA and without PPSPA groups differed as to the activities and routines of children and their families by age groups. The analyses were performed using the Statistical Package for the Social Sciences (SPSS), version 23, with a significance level of 0.05 as reference. The power of the independent Student’s *t*-test was 0.97, calculated from the sample size of the study (n total=916) by GPower 3.1 (Macintosh, Dusseldorf, Germany).

## RESULTS

The 916 participating children live in apartments (54%) or independent houses (46%). The majority (72.8%) does not have external space in the residence and 88% do not have internal space dedicated to performing PA. Before the social distancing period, 67.3% of children practiced scheduled PA at least twice a week.

Table 1 shows how social distancing has led to changes in children’s and their family habits, by age group. At all ages, there is a decrease in the performance of PA, an increase in the use of screens and family activities (Table 1).

**Table 1.** Changes in children’s and families’ routines during social distancing (parental report comparing different habits during social distancing with the pre-social distancing period) by children’s age groups (G1: 3 to 5 years, G2: 6 to 9 years, G3: 10 to 12 years).

Habit	Age Group	Much more (%)	More (%)	Neither more nor less (%)	Less (%)	Much less (%)
Sleep	G1	14 (4.3)	84 (25.9)	177 (54.6)	37 (11.4)	12 (3.7)
	G2	36 (8.9)	145 (36.1)	190 (47.3)	27 (6.7)	4 (1.0)
	G3	29 (15.3)	75 (39.5)	69 (36.3)	13 (6.8)	4 (2.1)
Physical activity	G1	2 (0.6)	9 (2.8)	36 (11.1)	132 (40.7)	145 (44.7)
	G2	3 (0.7)	14 (3.5)	37 (9.2)	130 (32.3)	218 (54.2)
	G3	3 (1.6)	7 (3.7)	18 (9.5)	57 (30)	105 (55.2)
Playtime spent on screens	G1	129 (39.8)	123 (38.0)	47 (14.5)	12 (3.7)	13 (4.0)
	G2	187(46.5)	139 (34.6)	53 (13.2)	15 (3.7)	8 (2.0)
	G3	92 (48.4)	72 (37.9)	17 (8.9)	6 (3.1)	3 (1.6)
Family activities	G1	55 (17.0)	175 (54.0)	63 (19.4)	19 (5.9)	12 (3.7)
	G2	70 (17.4)	209 (52.0)	93 (23.1)	18 (4.5)	12 (3.0)
	G3	30 (15.8)	91 (47.9)	43 (22.6)	11 (5.8)	15 (7.9)

Data expressed as absolute number of children (percentage).

Table 2 shows the results regarding the differences between the groups (PPSPA and no PPSPA) on the time spent by children doing different activities performed during the day. For all habits analyzed (intellectual activities, sleep time, playtime spent on screens, playtime with and without physical activity, physical activity, overall physical activity, and overall sedentary time) there were no differences between children who performed scheduled physical activity before the social distancing period and those who did not, in any of the age groups studied (Table 2).

**Table 2.** Mean, standard deviation and Student’s *t*-test results on the effect of previous practice of scheduled physical activity in groups of activities performed by children, as reported by parents (G1: 3 to 5 years, G2: 6 to 9 years, G3: 10 to 12 years).

	Group	PPSPA – Yes (%)	PPSPA – No (%)	Mean ± SD Yes	Mean ± SD No	Student’s t-test
Intellectual activity time (hours)	G1	71	29	1.8±2.1	1.7±1.8	p=0.797
	G2	81	19	2.0±2.3	2.3±2.3	p=0.270
	G3	84	16	2.1±2.3	1.4±1.6	p=0.154
Sleep time (hours)	G1	71	29	9.8±1.2	10.0±1.2	p=0.386
	G2	81	19	9.2±1.1	9.5±1.1	p=0.128
	G3	84	16	9.3±1.3	9.2±1.2	p=0.742
Playtime spent on screens (hours)	G1	71	29	3.9±2.3	3.7±2.0	p=0.593
	G2	81	19	4.3±2.2	4.1±2.5	p=0.490
	G3	84	16	5.0±2.5	5.4±2.8	p=0.473
Playtime without physical activity (hours)	G1	71	29	2.8±1.7	3.0±1.6	p=0.507
	G2	81	19	2.3±1.6	2.7±1.7	p=0.072
	G3	84	16	1.6±1.6	1.6±1.5	p=0.942
Playtime with physical activity (hours)	G1	71	29	1.2±1.1	1.3±1.3	p=0.728
	G2	81	19	0.8±0.9	0.7±0.9	p=0.290
	G3	84	16	0.5±0.8	0.6±0.9	p=0.872
Physical activity time (hours)	G1	71	29	0.7±1.1	0.6±0.8	p=0.457
	G2	81	19	0.5±0.7	0.5±0.9	p=0.552
	G3	84	16	0.5±0.7	0.5±0.8	p=0.857
Overall sedentary time (hours)	G1	71	29	8.4±3.2	8.4±3.1	p=0.863
	G2	81	19	8.7±3.4	9.2±3.0	p=0.238
	G3	84	16	8.8±3.4	8.5±3.6	p=0.676
Overall physical activity time (hours)	G1	71	29	1.9±1.7	1.9±1.7	p=0.834
	G2	81	19	1.3±1.4	1.1±1.4	p=0.309
	G3	84	16	1.0±1.2	1.0±1.1	p=0.819

PPSPA: previous practice of scheduled physical activity. %: percentage of children in a group with (Yes) or without (No) PPSPA.

## DISCUSSION

Social distancing promoted a decrease in activities involving PA and an increase in screen time among Brazilian children. These results are in line with other investigations carried out during the pandemic period, in different locations around the world, such as Bosnia and Herzegovina, the United States, Canada, Croatia, Italy, Brazil, Portugal, among others.^1–5,8,9,16,17^ The constraints imposed on the use of community and outdoor spaces led to more sedentary patterns,^1^ and the closure of schools and social distancing made it difficult to maintain the PA levels undertaken by children. In addition, regular school days are important to control healthy children’s habits. Overweight and obese children find it more difficult to control their healthy habits and weight in non-school periods, and the pandemic exacerbates the risk of weight gain.^17^ Socializing with other children can also be a motivational factor for carrying out group physical activity, which was impaired in this period.

Regular and structured PA and a healthy lifestyle are seen as facilitators for children to perform PA and display active behaviors.^18^ A systematic review reported that parents of children who perform regular and structured PA identify higher levels of PA in their children.^18^ However, results of the present study show that social distancing has similarly impacted the level of PA and other habits of Brazilian children, regardless of whether they had the habit of performing scheduled physical activity at least twice a week before the pandemic or not.

Sekulic et al. identified that Croatian adolescents with a higher fitness index before the restriction measures also showed a higher level of PA during the period of social distancing compared to adolescents with a lower level of PA before social distancing.^5^ However, the pandemic measures taken in Croatia and Brazil were different. The study reports that schools and parks were closed, but there was no prohibition on individual training, such as cycling and running.^5^ In Brazil, mobility was more restricted. With the greater spread of the virus and the number of infected and deceased people, a longer stay home period was adopted. Thus, children with PPSPA did not have the option of continuing their activities, as it was possible for some adolescents in Croatia. In addition, age can be a limiting factor in the continuity of scheduled physical practice, as adolescents already have greater autonomy to perform outdoor activities, if allowed in their region, and children up to 12 years are less autonomous and more dependent on their parents for mobility. Thus, the Brazilian social distancing period had a similar impact on the PA level of the children, with all of them having limited activities outside the home. In other countries, such as Canada, children from some regions were still able to carry out activities outside the home.^4^ Thus, this analysis may have different results depending on the location and measures adopted. In addition, the maintenance of structured practices normally requires the supervision and guidance of an adult, which does not occur adequately in social distancing, and these children are left without guidance for physical practice, often not knowing how to train by themselves and without suitable material.

Children usually get the recommended PA time by going to school, having physical education classes, playing sports, games, active play, and going to parks and playgrounds.^19^ During social distancing, all these opportunities were impaired. In other words, staying home did not only hinder the performance of programmed and structured PA practices, but also all the motor possibilities of regular children’s routines, which are important for reaching the recommended level of PA.^19^ Thus, keeping children physically active and with a healthy lifestyle during the pandemic is a challenge, as children have lost all of their usual routines and opportunities to be physically active. Therefore, it is clear that for a child to maintain a physically active life, all of their routines are important, not only scheduled practice of PA twice a week.

Low levels of physical activity in children have several negative health consequences, such as chronic diseases, weight gain, loss of muscle and cardiorespiratory fitness, psychosocial disorders, and lower academic performance.^1,3,19,20^ Being physically active during childhood and adolescence is also important because it is related to the maintenance of this habit into adulthood.^1^ In addition, higher levels of physical inactivity and homestay can generate vitamin D deficiency in these children.^21^ Children’s parents play a fundamental role in promoting experiences for their children that help them to maintain adequate levels of physical activity. In addition, the place where the child spends most of the day directly influences its physically active behavior.^1,4^ Understanding the family and home context during the pandemic is of fundamental importance because, during this period, children spent more time at home, where they were in greater contact with their parents and being influenced by their household characteristics and dynamics.

Having PA habits can help children to cope with social distancing periods, since higher levels of PA help to prevent mental illnesses, such as depression, stress, and anxiety, conditions that have been exacerbated during the pandemic.^15,22,23,24^ In addition, ensuring an adequate level of physical activity is a public health concern, as physical inactivity contributes to several chronic illnesses, and there may be negative economic and health consequences in the long term if the “new normal” includes great constraints to PA.^2,3,5,19^ For this reason, in possible future moments of new pandemics and social distancing, children’s PA should be encouraged. Several measures can be encouraged, such as creating online resources with suggestions to maintain and enhance PA, with exercises for each age group, creating online classes taught by specialized professionals, educating parents about the importance of stimulating their children’s PA, allowing children’s leisure outdoor venues to open, or reopen them as soon as possible if they had to be closed, and using technological devices, such as video games, that require body activity (Wii, Xbox, and others), promoting PA inside the home.^4,7,16^ The findings of the present study may contribute to the formulation of public policies for children during pandemic times.

This study is limited by the lack of investigation of the participants’ socioeconomic level, and by the different circulation restrictions imposed by federal, state, and municipal governments in Brazil. Future studies should address these different realities and investigate the effects of programs to stimulate PA and other healthy habits during social distancing.

In conclusion, the practice of scheduled physical activity before the social distancing imposed by COVID-19 did not influence the time of PA and other habits of Brazilian children during this period. Children with or without previous practice of physical activity had the same routines during social distancing. According to parental reports, there was a decrease in the time dedicated to PA during this period.
